# Coronary artery compression in percutaneous pulmonary valve implantation: a case series

**DOI:** 10.3389/fcvm.2026.1917017

**Published:** 2026-10-15

**Authors:** Rujira Buntharikpornpun, Supaporn Roymanee, Worakan Promphan, Phuoc Duong

**Affiliations:** 1 Division of Pediatric Cardiology, Department of Pediatrics, Faculty of Medicine, Prince of Songkla University, Hat Yai, Songkhla, Thailand; 2 Pediatric Heart Center, Department of Medical Services, Ministry of Public Health, Queen Sirikit National Institute of Child Health, Bangkok, Thailand; 3 Alder Hey Children’s Hospital, Liverpool, United Kingdom

**Keywords:** case report, coronary compression, percutaneous, pulmonary valve replacement, transcatheter

## Abstract

Catastrophic complications of coronary artery compression after percutaneous pulmonary valve implantation (PPVI) are uncommon in patients with a dysfunctional native right ventricular outflow tract after Tetralogy of Fallot repair (rTOF). Nonetheless, we report three rTOFs with a high risk of compromising coronary blood flow for PPVI. This risk has been elucidated by multimodal assessments, including robust pre-procedural cardiac computed tomography and meticulous balloon interrogation with selective coronary angiography.

## Introduction

Percutaneous pulmonary valve implantation (PPVI) is an effective, minimally invasive procedure for patients with dysfunctional right ventricular outflow tract (RVOT) lesions, such as tetralogy of Fallot, truncus arteriosus, transposition of great arteries, and congenital pulmonary stenosis, following surgical or interventional procedures. PPVI not only defers the timing of redo surgery but also extends conduit lifespan (1, 2). Despite its excellent outcome, however, complications remain a concern. Among these complications, coronary artery compression, though rare, is a potentially fatal one, occurring in 5%–10% of PPVI cases using a balloon expandable valve platform (3–5). Although this complication is still reported with self-expandable valve platforms, its incidence is generally believed to be lower, likely due to their reduced radial force compared to balloon-expandable valve counterparts, as many cohorts have reported zero incidence of coronary compression or myocardial infarction (6–13).

Anatomical diversities of the coronary artery arrangement in patients with RVOT dysfunction make assessment of compression by percutaneous valve prosthesis challenging. Therefore, a comprehensive assessment of RVOT anatomy and coronary artery patterns in relation to the selected valve is crucial for every patient. In high-risk cases, the result of simultaneous balloon interrogation and selective coronary angiography should be taken into account.

We report three cases of repaired tetralogy of Fallot (rTOF) with a potential risk of coronary artery compression following PPVI with self-expandable valves. In these cases, coronary artery compression was demonstrated on invasive testing during occlusion of the RVOT with a compliant balloon. Specifically, two cases had a preexisting anomalous coronary artery course of the right coronary artery, while one case experienced compression of the left main stem. Downsizing the valve with an alternative landing zone option allowed the patient to be saved from such serious complications. Furthermore, risk factors identified from prior imaging assessment correlated with the invasive testing. This suggests that such assessments can be used to predict potential complications before patients are taken to the catheter laboratory.

## Case report

### Case 1

A 20-year-old male with rTOF was scheduled for PPVI. This was indicated by cardiac magnetic resonance (CMR) results 13 years post-surgery, showing a pulmonary regurgitation fraction (PRF) of 56.1%, a right ventricular end-diastolic volume index (RVEDVi) of 157 mL/m^2^, and a left ventricular ejection fraction (LVEF) of 49%.

Pre-procedural cardiac computed tomography (CCT) revealed a previous valve-sparing repair, infundibular resection, and ventricular septal defect (VSD) closure. No transannular patching was evident in the CT study. A single coronary artery originated from the left/posteriorly facing sinus, giving rise to a left circumflex artery (LCx), left anterior descending artery (LAD), and two separate branches of the right coronary artery (RCA). The marginal branch of the RCA followed a retropulmonary course, looping anteriorly to the main pulmonary artery (MPA) trunk along the valve annular groove and coursing towards the right ventricular free wall. The second branch of the RCA system followed a retroaortic course towards the right atrioventricular groove (Figure 1A). The distance from the RVOT landing zone to coronary artery and to the chest wall are 1.5 mm and 26.8 mm, respectively.

**Figure 1:**
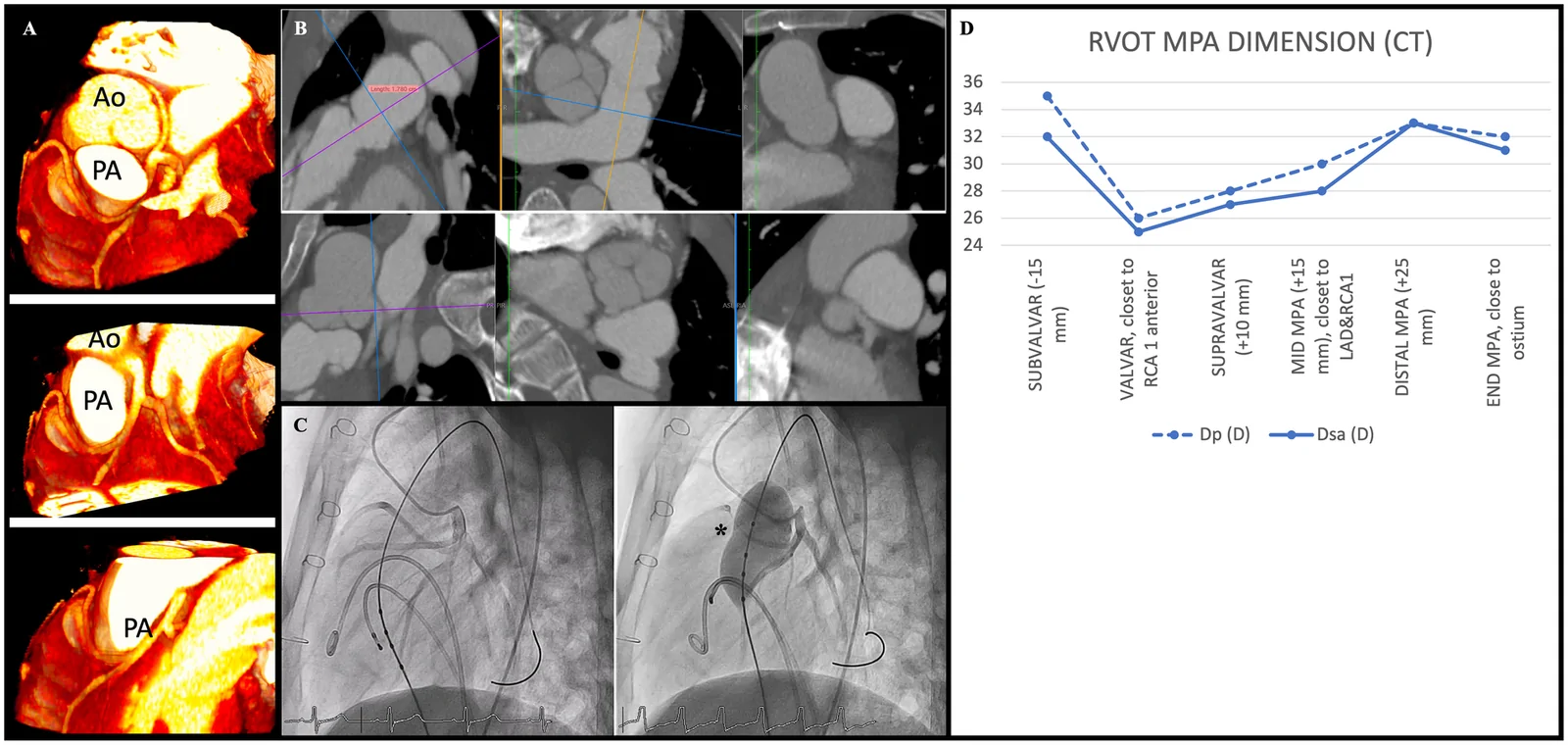
**(A)** CT showed a single coronary artery arising from the left posterior-facing sinus. The marginal branch of the RCA follows a retropulmonary course, and the second branch of the RCA system follows a retroaortic course. **(B)** CT showed the relation of the coronary artery to the RVOT. **(C)** The RCA (asterisk) flow was significantly diminished during simultaneous balloon interrogation and selective coronary angiography. **(D)** Diameter plot profile in diastole. *Dp* Perimeter derived diameter, *Dsa* Surface area derived diameter, ***(D)*** Diastole.

Initially, based on CCT measurements, the intended device was a 28–25 mm VenusP valve (Venus Medtech, Hangzhou, China). A diameter plot profile in diastole (showing larger valvar/subinfundibular regions) indicated a diameter of 25–26 mm, derived by perimeter and surface area, at the level of the valve annulus where the coronary course was closest (Figures 1B,D). This coincided with the expected waist on balloon sizing and the area of highest risk for coronary compression. The mid and distal segments of the MPA were also in contact with the coronary artery, though with a greater clearance distance.

During balloon sizing, a selective coronary angiogram with simultaneous RVOT balloon occlusion using a 30-mm PTSX balloon catheter (B. Braun Interventional Systems Inc., Pennsylvania, USA) was performed. This revealed RCA compression anterior to the RVOT, at a waist size of 21–23 mm (Figure 1C). Consequently, the procedure was abandoned.

### Case 2

A 21-year-old male with rTOF was indicated for PPVI based on CMR criteria 15 years post-surgery. These criteria included a PRF of 45.3%, RVEDVi of 173 mL/m^2^, right ventricular end-systolic volume index (RVESVi) of 124 mL/m^2^, right ventricular ejection fraction (RVEF) of 29%, and LVEF of 37%.

Pre-procedural CCT showed a single coronary artery system originating from the left/posterior-facing sinus. The coronary artery followed a retropulmonary course, giving rise to the left coronary artery (LCA) and RCA system, with the entire course of the RCA anomalously positioned anterior to the RVOT, closest to the pulmonary valve annulus (Figure 2A). Operation notes were unavailable. CCT findings indicated infundibular resection, sparing the pulmonary valve annulus. The distance from the RVOT landing zone to coronary artery and to the chest wall are 2.7 mm and 15.9 mm, respectively.

**Figure 2:**
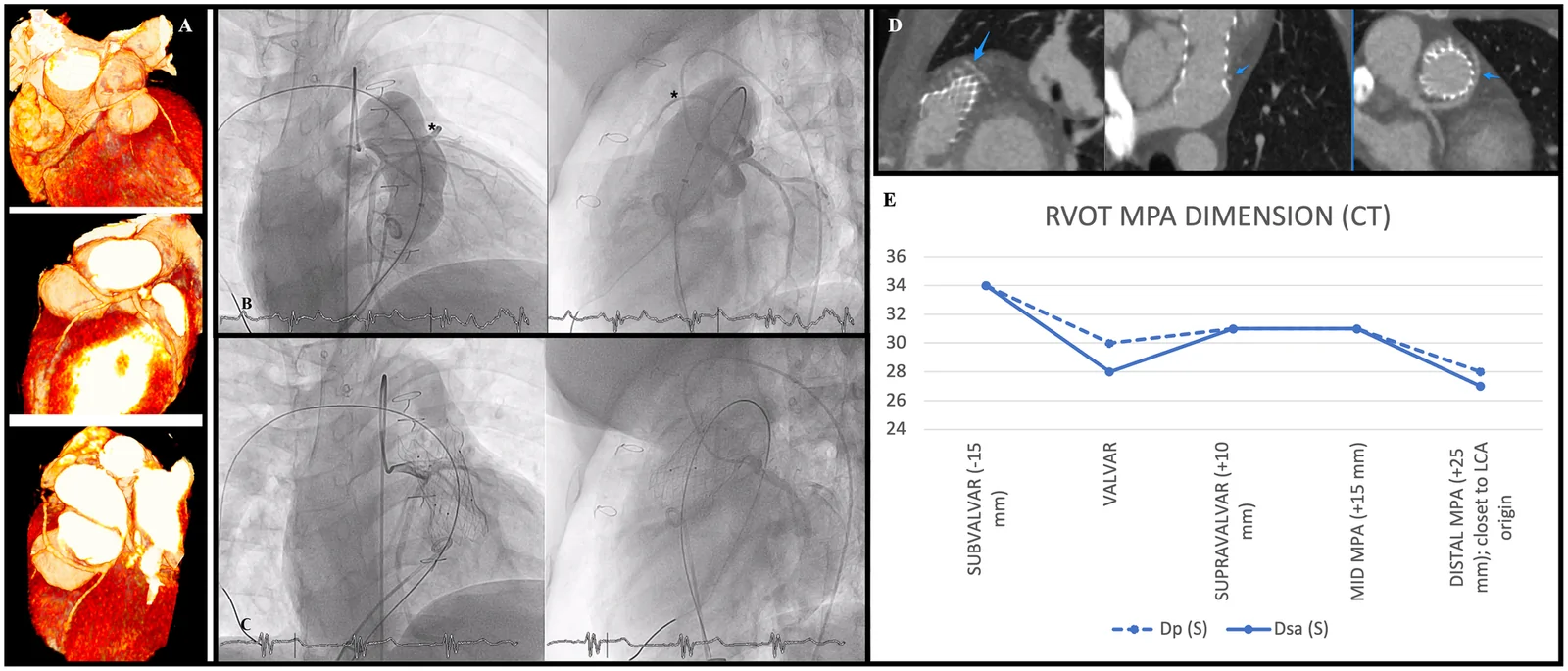
**(A)** CT revealed a single coronary artery system running a retropulmonary course. **(B)** A selective LCA angiogram simultaneously with balloon interrogation showing mild impairment of RCA (asterisk) flow. **(C)** After the 28-mm Venus *P* valve was deployed, a selective LCA angiogram showed no RCA compression. **(D)** CT 3 months post-procedure. **(E)** Diameter plot profile in systole. *Dp* Perimeter derived diameter, *Dsa* Surface area derived diameter, ***(S)*** Systole.

A diameter plot from systolic acquisition (Figure 2E) showed a maximal diameter of 27–28 mm at the area of closest main coronary course posteriorly (distal MPA) and up to 28–30 mm at the closest anterior course of the RCA. Balloon interrogation, performed with a 34-mm Amplatzer ASD sizing balloon catheter (Abbott, USA), demonstrated a reduction in distal RCA flow after full inflation of the balloon with 60 mL of diluted contrast media (Figure 2B). The balloon waist diameter measured 28.9 mm and 29.3 mm in two planes.

As the valve annular measurement during balloon interrogation matched the CCT planning, we assumed reduced compliance at this level, which would enable good anchoring points for valve conformation without oversizing. The clearance in the infundibulum allowed the proximal flare to conform and hold, while the muscular infundibulum portion below lowered the risk of proximal valve embolization. We opted not to oversize, choosing a valve matching the nominal size of the valve annulus, with the plan to rely on the radial force of the flare for good landing.

PPVI was performed with a self-expandable VenusP valve measuring 28–25 mm (Venus Medtech, Hangzhou, China) through a 26 Fr Gore Dryseal sheath measuring 65 cm (W.L. Gore & Associates, Inc., Arizona, USA), which was also used as a valve retriever in case of coronary compression. A coronary angiogram post-implantation showed normal flow to the distal RCA (Figure 2C). Follow-up CCT at 3 months showed the cylindrical portion of the valve measured 28 mm in diameter (as per design standard), with conformation to 50% of the perimeter at the level of the original pulmonary valve annulus, where the RCA crossed over. The RCA caliber was uniform along the course of the implanted valve, with good contrast perfusion to the distal segments (Figure 2D). The patient remained asymptomatic, and CMR showed no evidence of coronary compression at the 3-year follow-up.

### Case 3

A 39-year-old male with rTOF presented with severe PR. PPVI was indicated based on CMR criteria (PRF of 46%, RVEDVi of 167 mL/m^2^, and LVEF of 48%) and symptoms of dyspnea.

Pre-procedural CT showed a transannular patch repair, with the patch extending to the mid-MPA and significant patch calcification, occupying up to 25% of the perimeter at the valvar level (Figure 3A). The distance from the RVOT landing zone to coronary artery and to the chest wall are 2 mm and 8.3 mm, respectively. The MPA was “folded,” causing the distal MPA to form an almost 90-degree angle to the RVOT. The right pulmonary artery was significantly dilated compared to the left pulmonary artery (LPA) origin stenosis. In the left anterior oblique/cranial view, there was concern that a conventional transannular position, given the RVOT/MPA geometry, might cause the valve to protrude too low into the RVOT, posing a risk of ventricular dysrhythmias. *In vitro* simulation on a 3D-printed model built from the patient's CCT confirmed this possibility. The technology was provided by HARN Engineering Solutions, which fabricated the model using Acrylonitrile Butadiene Styrene (ABS), a rigid filament. With this model, we placed the VenusP 30–25 mm valve in the annular and supra-annular positions. At the annular position, the distal flare was at the RVOT close to the coronary artery while the distal flare was much farther from the coronary artery in the supra-annular position. This experiment supported a geometrical assessment of valve positioning, but could not be used to predict the actual mechanical risk of coronary compression. We opted for a supra-annular position with a shorter valve to avoid blocking access to the LPA for future stenting.

**Figure 3:**
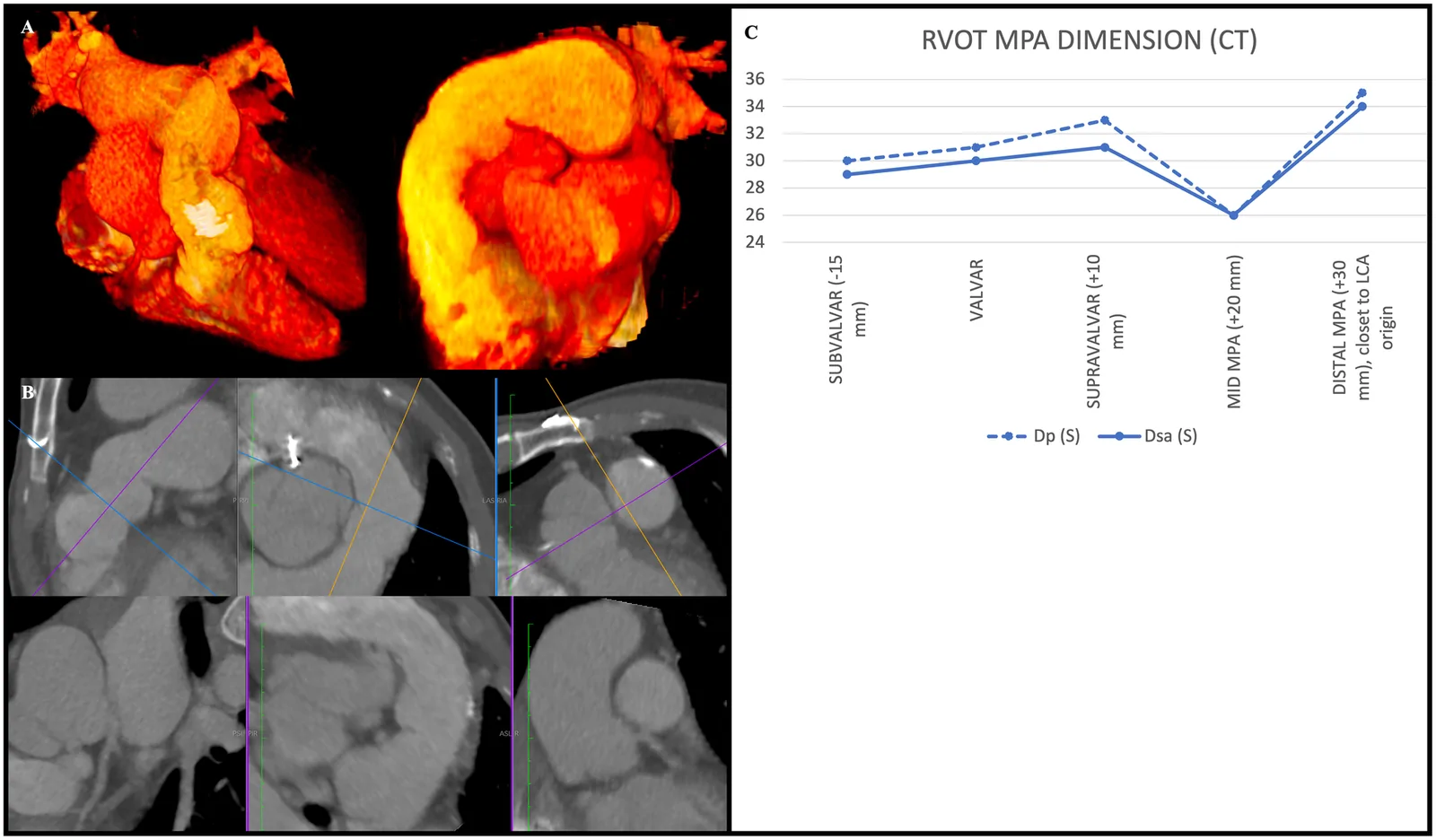
**(A)** pre-procedural CT showing a horizontal RVOT. **(B)** The LCA origin is close to the distal MPA. **(C)** Diameter plot profile in systole. *Dp* Perimeter derived diameter, *Dsa* Surface area derived diameter, ***(S)*** Systole.

Additionally, the aortic root was dilated (43 × 47 × 48 mm), with clockwise rotated coronary ostia and a normal proximal course of the coronary arteries. We identified the distal MPA to be in close contact with the main stem of the LCA (Figure 3B), which was considered a contravening factor for distal valve landing.

Balloon interrogation using a 34-mm Amplatzer ASD sizing balloon catheter (Abbott, USA) demonstrated reduced flow to the left coronary artery at the LCA main stem, distal to the mid-MPA fold, corresponding to CT findings (waist at mid–MPA fold: 24 × 25 mm, matching CT measurements of 25 × 25 mm) (Figure 4A). Similar to Case 2, the region in closest proximity to the coronary artery tolerated a 34–35 mm diameter on CT imaging and 32–34 mm during balloon sizing. As the CT measurement matched the balloon sizing at the mid-MPA fold, the area was adequately less compliant to ensure proper valve conformation/contact without significant oversizing. It also allowed the closest contact point to the coronary artery to remain at its native maximal diameter. We opted for a VenusP 30–25 mm valve (Venus Medtech, Hangzhou, China). No ischemic ECG changes or ventricular dysrhythmias were observed during the procedure. Follow-up CT at 2 months showed a valve diameter of 29 × 31 mm and unobstructed flow to the LCA on all dynamic phases (Figure 4B). The patient remained asymptomatic, and CMR revealed no evidence of coronary compression at the 3-year follow-up.

**Figure 4:**
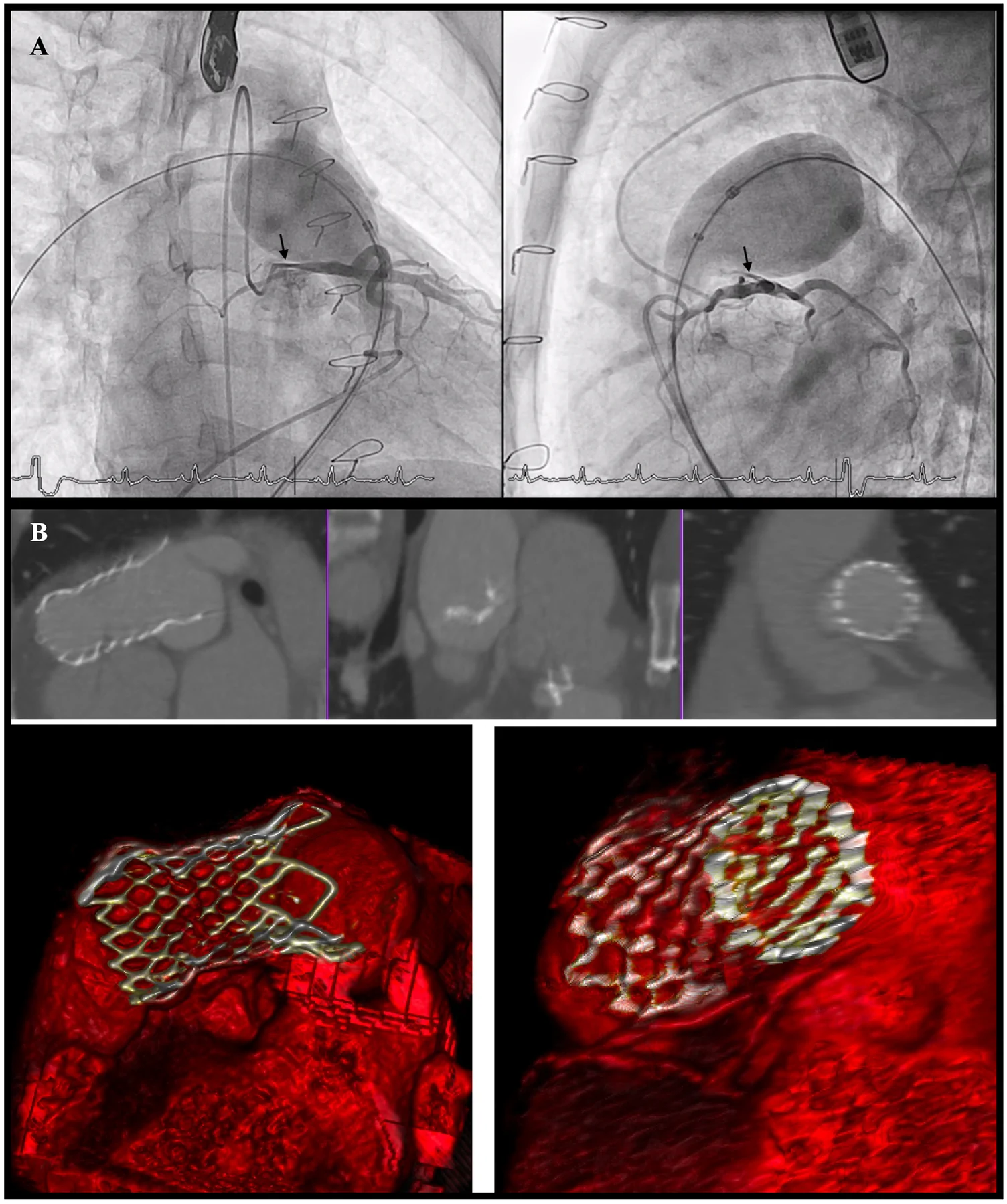
**(A)** balloon interrogation with a 34-mm amplatzer ASD sizing balloon catheter (Abbott, USA) demonstrated reduced flow to the left coronary at the LCA main stem (arrow). **(B)** CT 2 months post-procedure.

## Discussion

PPVI is a safe and effective procedure designed to reduce the need for re-operation and minimize recovery time. However, achieving optimal outcomes and preventing major adverse events requires a comprehensive understanding of RVOT morphology, coronary artery patterns, procedural steps, and available valve options.

Coronary artery compression, one of the most serious complications of PPVI, has been reported in 5%–10% of cases (3–5). Although pre-procedural imaging currently aids in predicting this problem, it remains a difficult and complex issue. In the era of self-expandable valves, coronary compression is less understood. While it is logical to assume that coronary artery compression is less common because of the lower radial force of self-expandable valves, we have demonstrated that this rare complication still manifests in both normal and abnormal coronary arrangements. In this paper, we also show that careful study of pre-procedural images plays an important role in mitigating risks and potentially converting risks into procedural successes.

Several factors can be derived from CT/MRI imaging tailored toward intervention:

1. Coronary artery anatomy, origin, course, closest point of contact, and associated risk factors (dilated aortic root, extreme clockwise rotation).

In patients undergoing PPVI, abnormal coronary artery anatomy significantly increased the risk of coronary artery compression (22% versus 1.8%) (4). Overall, 71% of patients who experienced compression had abnormal anatomy, compared to only 14% of those without compression. It is worth noting that RVOT conduits accounted for two-thirds of the study population and that balloon-expandable valves were used in all patients. This factor should be taken into account when evaluating self-expandable valve platform, as despite having lower radial force, it typically requires oversized valve implantation. In our report, the first two cases were rTOF patients with abnormal coronary patterns. Both cases showed evidence of coronary artery compression during balloon testing. Nonetheless, this problem did not preclude successful PPVI in the second case, as the patient's tubular RVOT anatomy with a distinct waist allowed us to select a valve that was not oversized. Thus, the valve could be positioned in the intended RVOT without causing any compression on the coronary arteries.

Currently, many practices perform multi-modal imaging, including echocardiogram, CCT, CMR imaging, and balloon testing, prior to PPVI. Various approaches have been proposed to identify coronary artery compression; however, a universally accepted standard protocol is still under development. CCT is frequently chosen as the imaging modality for assessing RVOT morphology, PA branch anatomy, coronary artery patterns, and their relationship to the RVOT (14).

Despite the absence of a recommended safe distance between the RVOT and coronary artery—owing to factors such as calcification, scar tissue, and residual conduits or stents that may result in an incompressible mass causing coronary artery compression—it is important to measure the distance between the RVOT landing zone and the coronary arteries. The study by Tezza et al. proposed that a critical indicator of the risk of coronary compression from imaging is the close proximity (<2 mm) of the coronary arteries to the RVOT or conduit (15). Furthermore, Rinaldi's study demonstrated that an RVOT landing zone distance of 3 mm to a coronary artery (*P* < 0.001), a landing zone distance of 8 mm to the chest wall (*P* = 0.026), and the presence of anomalous coronary arteries (*P* = 0.003) are associated with an increased risk of coronary complications (16). More recently, the study by Romans et al. presented two accessible CMR measurements for predicting coronary artery compression (17). The first measurement is the shortest orthogonal distance from a coronary artery to the conduit, which is ≤2.1 mm. This criterion exhibited a sensitivity of 58% and a specificity of 73% in predicting coronary artery obstruction. The second measurement is the distance from the most stenotic area of the conduit to the coronary artery ≤13.1 mm. This criterion demonstrated a sensitivity of 83% and a specificity of 55%. If either criterion was met, the sensitivity was 92% and the specificity was 40%; however, if both criteria were met, the sensitivity decreased to 46%, while the specificity increased to 90%. In our study, the distance between the RVOT landing zone and the chest wall was >8 mm, whereas the distance to the coronary was <3 mm in all three cases (1.5, 2.7, and 2.0 mm in Cases 1, 2, and 3, respectively). Following the method described by Rinaldi et al., the proposed valve landing zone in the RVOT and the nearest coursing coronary arteries were identified, measured across axial, sagittal, and coronal views, and the shortest distance was recorded. In balloon-expandable valve platform deployed in RVOT conduits, the coronary compression is more challenging to predict due to uncertainty regarding the extent and direction of conduit expansion, the stiffness of calcified walls, and limited retrosternal space compared to self-expandable platforms in native RVOT anatomy. Therefore, the safety margins established in previous studies for balloon expandable valves may not directly apply to self-expandable devices. These three cases may provide preliminary hypotheses that CT-derived distance thresholds could have different predictive implications for balloon-test positivity and actual post-implantation coronary compression for self-expandable devices.

2. Diameter at different segments, dimensional distensibility, and landing zone planning.

Currently, there is no existing data regarding the risk of coronary displacement/compression in PPVI for self-expandable valves. Therefore, it is essential to understand the differences in PPVI planning between self-expandable and balloon-expandable valves. Self-expandable valves exert less radial force and are generally believed to be more forgiving in altering the maximal dimension of the RVOT/MPA.

Determining the maximal level of expansion in a structure with varying compliance at different segments requires careful planning and understanding. Expert-level advice relies on cumulative experience with balloon testing and understanding how pre-procedural imaging measurements translate to intra-procedural findings. Morphology is a key factor in securing valve stability and acts as the foundation for valve shape design and landing zone planning. We have learned that measuring the valve at different levels (subvalvar, annulus, sinus, sino-tubular junction/mid MPA, distal MPA) correlates with the shape of the valve design. Consequently, landing zone and sizing can be deduced from pre-procedural imaging. The dimensional distensibility can be inferred from these measurements. For instance, an RVOT landing zone with similar measurements during both systole and diastole, or on CCT compared to balloon sizing, suggests a non-compliant area. This yields a more promising outcome when implanting a non-oversized valve. Determining the largest possible diameter of the valve segment at the level of the closest course of the coronary artery and matching it with the nominal size of the chosen valve is the rationale we use in practice. It is essential to perform a balloon occlusion test with simultaneous coronary angiography to validate procedural planning and simulate valve radial force, especially in high-risk patients (5). However, some might argue that balloon sizing may have a false positive rate for the likelihood of coronary artery compression owing to balloon dilation, stenting, or PPVI, which can all potentially change the geometry or size of the RVOT conduit and alter the anatomic relationship of the RVOT with surrounding structures (18). Although the simultaneous balloon interrogation and selective coronary angiography showed coronary compression in all three cases in our studies, we considered proceeding in Cases 2 and 3. The main factor in Case 2 was the possibility of non-oversized valve implantation due to the RVOT size from CCT and balloon sizing measurements were similar assuming low dimensional distensibility at that area. In Case 3, the consideration point is the decision to place the valve in the supra-annular position which could avoid the distal flare to compress the RVOT where the coronary artery is close to and the RVOT size from CCT and balloon sizing at the mid MPA were similar which allowed us to select a non-oversized valve. In contrast, in Case 1, there was significant coronary compression during balloon interrogation. The RVOT landing zone measured 25–26 mm on CCT but only 21–23 mm on balloon sizing which was measured where the RVOT is closest to the first RCA branch. This discrepancy between the CCT and balloon sizing measurements would have required implanting an oversized valve. Furthermore, deploying the valve in an alternative position was not feasible because the RVOT was also closed to the LAD at the supra-annular level. Therefore, the team decided against proceeding with the intervention in this case. In our experiences, patients with evidence of coronary compression during balloon interrogation should not be automatically excluded from PPVI if several criteria are met: first, the landing zone has a distinct, fixed waist or low distensibility (demonstrated by concordant systolic/diastolic CCT and balloon sizing measurements that allow non-oversized valve selection; second, a supra-annular position is anatomically viable; and third, alternative valve designs (such as flare-less stents) can avoid vulnerable compression zones identified on pre-procedural imaging. In addition to established risk factors derived from balloon-expandable valves in RVOT conduits, these considerations may guide decision-making for self-expandable valve platforms. However, these hypotheses will need to be evaluated more thoroughly in larger future studies.

In summary, coronary artery compression is a serious and potentially fatal complication in PPVI. It is crucial for operators to be fully aware of this complication, and further studies are necessary to establish a standardized approach to effectively prevent and avoid it (14, 19, 20). Various factors contribute to the occurrence of coronary artery compression, including the anatomy of the RVOT, the presence of abnormal coronary arteries, the relationship between the RVOT and the coronary course, and the coronary angiogram with simultaneous balloon interrogation. By considering these factors, the risk of coronary artery compression during PPVI can be minimized.

## Conclusion

PPVI is a safe and less invasive procedure for treating patients with RVOT lesions. However, the potentially life-threatening complication of coronary artery compression is a persistent concern, as it can occur regardless of normal or abnormal coronary artery patterns. Vigilant multi-modality imaging evaluation and operator awareness play pivotal roles in identifying high-risk patients and proactively preventing these complications.

## Data Availability

The raw data supporting the conclusions of this article will be made available by the authors, without undue reservation.
